# Nurse Coaching and Mobile Health Compared With Usual Care to Improve Diabetes Self-Efficacy for Persons With Type 2 Diabetes: Randomized Controlled Trial

**DOI:** 10.2196/16665

**Published:** 2020-03-02

**Authors:** Heather M Young, Sheridan Miyamoto, Madan Dharmar, Yajarayma Tang-Feldman

**Affiliations:** 1 Betty Irene Moore School of Nursing University of California, Davis Sacramento, CA United States; 2 College of Nursing The Pennsylvania State University University Park, PA United States

**Keywords:** mHealth, electronic health record, type 2 diabetes, motivational interviewing

## Abstract

**Background:**

Type 2 diabetes is a growing public health problem amenable to prevention and health promotion. As healthy behaviors have an impact on disease outcomes, approaches to support and sustain diabetes self-management are vital.

**Objective:**

This study aimed to evaluate the effectiveness of a nurse coaching program using motivational interviewing paired with mobile health (mHealth) technology on diabetes self-efficacy and self-management for persons with type 2 diabetes.

**Methods:**

This randomized controlled trial compared usual care with an intervention that entailed nurse health coaching and mHealth technology to track patient-generated health data and integrate these data into an electronic health record. The inclusion criteria were as follows: (1) enrolled at 1 of 3 primary care clinics, (2) aged 18 years or above, (3) living with type 2 diabetes, and (4) English-speaking. We collected outcome measures at baseline, 3 months, and 9 months. The primary outcome was diabetes self-efficacy; secondary outcomes were depressive symptoms, perceived stress, physical functioning, and emotional distress and anxiety. Linear regression mixed modeling estimated the population trends and individual differences in change.

**Results:**

We enrolled 319 participants; 287 participants completed the study (155 control and 132 intervention). The participants in the intervention group had significant improvements in diabetes self-efficacy (Diabetes Empowerment Scale, 0.34; 95% CI –0.15,0.53; *P*<.01) and a decrease in depressive symptoms compared with usual care at 3 months (Patient Health Questionnaire-9; 0.89; 95% CI 0.01-1.77; *P*=.05), with no differences in the other outcomes. The differences in self-efficacy and depression scores between the 2 arms at 9 months were not sustained. The participants in the intervention group demonstrated a significant increase in physical activity (from 23,770 steps per week to 39,167 steps per week at 3 months and 32,601 per week at 9 months).

**Conclusions:**

We demonstrated the short-term effectiveness of this intervention; however, by 9 months, although physical activity remained above the baseline, the improvements in self-efficacy were not sustained. Further research should evaluate the minimum dose of coaching required to continue progress after active intervention and the potential of technology to provide effective ongoing automated reinforcement for behavior change.

**Trial Registration:**

ClinicalTrials.gov NCT02672176; https://clinicaltrials.gov/ct2/show/NCT02672176

## Introduction

### Background

Type 2 diabetes is a growing public health problem amenable to prevention and health promotion [1]. The prevalence of diabetes in the United States will increase from 9.3% in 2012 to an estimated 25% to 28% by 2050, with type 2 diabetes accounting for 90% to 95% of cases [2]. Physical inactivity, poor eating habits, obesity, and smoking are common risk factors for type 2 diabetes. The connections between health behavior and disease outcomes indicate the importance of a patient-centered, proactive, and evidence-based approach to prioritizing and enacting lifestyle choices [3].

Having a chronic condition has implications for all aspects of daily life as the individual navigates choices about nutrition, physical activity, sleep, stress management, and medication regimen. Bandura and Adams [4] established that self-efficacy, the belief in one’s ability to influence events, can effect changes in behavior. For decades, researchers and clinicians have recognized the importance of perceived self-efficacy in the management of diabetes, including the ability of individuals to make healthy lifestyle decisions, adhere to medication and treatment regimens, and manage stress [5]. Encounters with health care providers are episodic and usually focus on monitoring and adjusting medical treatment. As optimal health in diabetes requires a more active approach by individuals to self-manage their condition and to engage in lifestyle behavioral changes, health care providers could contribute to better outcomes by offering personalized support.

Diabetes education programs and group classes may be effective in the short term but appear to be insufficient to sustain behavioral changes (eg, improvements in physical activity and healthy eating) and self-management skills [6,7]. Motivational interviewing (MI) and health coaching have the potential to customize strategies according to the individual’s priorities and interests. MI is a counseling approach to build capacity to solve problems, improve self-efficacy, and support behavioral change in diabetes management [8-11]. Health coaching utilizes MI concepts to facilitate behavior change by encouraging individuals to establish attainable personal goals, brainstorm strategies to achieve goals, and self-monitor behaviors, all within the context of an interpersonal relationship with a coach [12,13]. The results of a systematic review on health coaching found improved physiological, behavioral, psychological, and social outcomes in people with chronic conditions [14]. Qualitative exploration of patient perspectives on unmet needs in self-management revealed gaps in the existing programs in their ability to support emotional regulation, psychological adjustment, and behavior change [15]. Our group previously demonstrated the effectiveness of MI and health coaching in sustaining diabetes self-efficacy in rural communities [16]. These interventions typically rely on self-report of lifestyle changes, such as diet or physical activity modifications, limiting precision in quantifying behavioral improvements.

The International Diabetes Federation outlines clinical guidelines for type 2 diabetes management, including educating patients and providers, setting goals for self-management of blood glucose, generating a structured profile, providing feedback to patients about their results, using these data to modify treatments, and engaging in shared decision making [17]. Despite these guidelines, a systematic review of type 2 diabetes management indicates that these principles have not been widely adopted in primary care. One of the gaps is that diabetes self-management education and support programs incorporating mobile health (mHealth) inconsistently integrate data and feedback to change treatment and support behavior change [18].

Mobile technology offers new opportunities to track health behaviors, provide reinforcement through immediate feedback about objective measures of behavior such as steps taken, and improve health outcomes in chronic diseases [19-21]. Technology-enabled diabetes self-management solutions with a feedback loop using patient-generated health data (PGHD) to tailor education and individualize feedback improved hemoglobin A_1C_ [22-25]. Wearable tracking devices and mHealth apps that capture health behaviors offer an objective view of daily activity, which was not previously available [26]. These technological advances become more salient when PGHD are part of the clinical record, incorporated into the care plan of adults with type 2 diabetes, offering access to data for clinicians and improved precision health opportunities.

### Objective

This study examined the impact of a novel intervention using MI-based nurse health coaching combined with wearable activity trackers that integrate patient-generated activity data into the patient’s electronic health record (EHR) to improve health among adults with type 2 diabetes. We hypothesized that individuals randomized to the intervention group would show overall improved self-efficacy compared with individuals in the usual care group.

## Methods

### Study Design

A detailed description of the study design was previously reported in a clinical trial protocol [27]. This was a randomized controlled trial with 2 arms: (1) usual care through primary care and (2) the Patient and Provider Engagement and Empowerment Through Technology (P^2^E^2^T^2^) Program—nurse coaching paired with mobile sensor technology. The study was approved by the institutional review board of the University of California, Davis, and registered at ClinicalTrials.gov (NCT02672176).

### Recruitment

We recruited participants from 2 suburban and 1 urban primary care clinic within an academic health center in Northern California. The inclusion criteria were as follows: (1) aged 18 years or above, (2) receiving care at 1 of the 3 clinics, (3) living with type 2 diabetes and having HbA_1c_ of 6.5% (48 mmol/mol) or higher, and (4) able to speak English. Participants were ineligible if they did not have access to a telephone, were not able to consent because of cognitive impairment, or were pregnant. Our power analysis determined that with 100 participants per arm, we would have 80% power to detect differences in self-efficacy. We queried the health system EHR and diabetes registry to identify eligible participants who subsequently received mailed letters and telephone calls. Potential participants were told that the study focused on using enabling technology to support their health in diabetes. Study data were collected and managed using Research Electronic Data Capture tools hosted at the Clinical Translational Science Center at UC Davis [28,29]. REDCap (Research Electronic Data Capture) is a secure, Web-based software platform designed to support data capture for research studies, providing (1) an intuitive interface for validated data capture, (2) audit trails for tracking data manipulation and export procedures, (3) automated export procedures for seamless data downloads to common statistical packages, and (4) procedures for data integration and interoperability with external sources. Following telephone consent, we used Research Electronic Data Capture to randomize participants to 1 of the 2 groups in a 1:1 ratio, stratified by clinic site, to either the usual care (control group) or the P^2^E^2^T^2^ program. We used stratified block randomization to ensure a balanced number across the 2 groups within each site. The participants completed written consent during their onboarding session. Participants and research team members not involved in recruitment were blind to the randomization.

### Clinical Trial of Patient and Provider Engagement and Empowerment Through Technology

#### Usual Care

Participants in this group received usual care through their primary care clinic. Usual care comprised standard health care visits with providers and access to classes, resources, and services (ie, diabetes management and weight loss education, electronic learning videos, and care coordination). At the orientation meeting, the study team members provided instruction on how to access these resources and services as well as how to use the health system’s patient portal (MyChart).

#### Patient and Provider Engagement and Empowerment Through Technology Intervention Program Group

The intervention group participants received the same care through their primary care clinic and training as those receiving usual care regarding health system services and resources. In addition, the intervention included (1) nurse health coaching and (2) mHealth technology to track PGHD and integrate these data into the EHR (see Multimedia Appendix 1).

#### Nurse Health Coaching

The nurse health coaches for the intervention were 3 registered nurses (RNs) with experience in both health coaching and management of chronic disease. To promote fidelity to the intervention and a common approach to coaching participants, the nurses received core training in MI-based coaching and diabetes management. All the RN health coaches delivering the intervention completed the HealthSciences Institute’s Registered Health Coach (RHC) and Chronic Care Professional training programs (www.healthsciences.org). A final performance evaluation using the Motivational Interviewing Treatment Integrity (MITI) 3.1.1 global scale evaluation tool confirmed health coaching competency before the receipt of the RHC certificate [30]. Nurses also completed a refresher course in diabetes management through the American Association of Diabetes Educators as well as the standard health system orientation on policies, procedures, and EHR training.

We paired each participant with a nurse health coach who delivered 6 individual sessions using a counseling style based on the concepts of MI. Sessions were structured to promote mutual goal setting, enhance self-efficacy in health behavior change, and assist individuals to derive meaning from data to reinforce choices and behaviors. Two RN researchers with nurse coaching experience in diabetes audited 8 of the 158 (5%) of the participant sessions and scored the coach using the MITI. They provided timely feedback to the coaches during weekly debriefing sessions, reviewed scores, and discussed optimization strategies by reviewing scenarios.

The participants had an in-person orientation with the nurse coach, followed by telephone sessions every 2 weeks for 3 months (6 contacts total). The initial MI session elicited motivations and set goals with tracking metrics to gauge the progress toward goals at subsequent sessions. Throughout the sessions, the coaches encouraged the participants to identify facilitators and barriers to achieving their health goals.

#### Mobile Health Technology and Integration of Patient-Generated Health Data Into the Electronic Health Record

We provided a wearable tracking device (initially, Basis Peak, then Garmin VivoSmart Heart Rate [HR]) to the intervention group participants. This device generated real-time information about steps taken, distance walked, active minutes, heart rate, and hours of sleep at night and synced the data to either an iPhone operating system mobile phone and/or iPod Touch. We provided the iPod Touch to participants who did not already possess this technology. We preinstalled MyFitnessPal, a mobile app, on the devices to allow participants to log and track nutritional consumption if they chose. We provided in-person or telephonic technical support to all participants—including the usual care group participants—throughout the duration of the study. We encouraged the participants to wear the activity tracker for the entire 9-month duration of the study.

PGHD were integrated into the EHR when participants performed regular synchronization of the activity tracker to their personal device. We used 2 connectors, Apple HealthKit and MyChart, to accomplish the automatic transmission of data to the EHR. We used Synopsis, a feature within the EHR, to design a single screen page of relevant PGHD along with clinically relevant data elements (ie, laboratory values and medications). In the case management module of Epic Electronic Health Record, we designed a summary documentation form for the nurse coaching sessions. We sent a final summary of goals and achievements to the primary care providers. Using these tools, the participants, providers, and nurse health coaches could view trends in activity levels, sleep, and nutritional intake on either their smart device or on a computer.

### Changes After Trial Commencement

Early in the intervention period, we experienced an unexpected recall of the Basis Peak activity tracking device because of a safety issue that required identifying and selecting a replacement device. The study team, in collaboration with the advisory boards, worked diligently and promptly to identify, test, and select a replacement (Garmin VivoSmart HR) and then distribute the new device to the participants in the intervention arm of the study, providing technical support to these participants as needed. This recall affected 79 participants; most of these participants received and were oriented to their new devices within 2 weeks of the recall.

### Measures

The participants completed Web-based surveys at baseline, 3 months (coinciding with the end of the intervention or 3 months from baseline), and 9 months. The baseline survey included demographic information (age, gender, race and ethnicity, education, income, and insurance type), health information (common chronic illness and health status), and technology use and adoption information. Readiness to change was assessed with 2 items measuring intention, *I am intending to make changes in my diabetes self-care in the next 6 months* and *I am intending to make changes in my diabetes self-care in the next month*, and then categorized into 3 groups: precontemplators (do not intend to make changes), contemplators (intend to act in 6 months), and preparers (intend to make a change in the next month) [31]. Surveys at all 3 time points assessed study outcome measures.

#### Primary Study Outcome

Diabetes self-efficacy (Diabetes Empowerment Scale [DES]–Short Form) [32] is an 8-item Likert-scale survey instrument that measures diabetes-related psychosocial self-efficacy. The overall score is the sum of scores of the 8 questions in the survey, with higher scores indicating greater self-efficacy.

#### Secondary Outcomes

Depression severity (Patient Health Questionnaire-9 (PHQ-9) [33] is a 9-question validated survey that measures the presence and severity of depression. The score is a sum of all the responses and ranges from 0 to 27. A score of 10 or above suggests the presence of depression.

#### Other Measures

The surveys also included Patient-Reported Outcomes Measurement Information System (PROMIS) [34] measures (physical function and emotional distress and anxiety) and the Perceived Stress Scale (PSS) [35]. The PROMIS physical function 4-item instrument assesses the current physical function in the individual. The PROMIS emotional distress and anxiety 4-item instrument measures self-reported fear, anxious misery, and hyperarousal symptoms. The PSS is a 4-item instrument that measures the degree to which situations in one's life are determined as stressful. We evaluated physical activity data measured as steps in the intervention group (who had the activity tracker). We audited the use of MyChart features for all participants. For the intervention group, the nurse coaches recorded goals and perception of goal attainment on the part of both the participant and the nurse coach.

### Statistical Methods

Descriptive analysis yielded means and SDs for continuous variables and frequencies for categorical variables. We examined distributions and collinearity to determine whether the data met the assumptions for planned statistical analyses. We compared the demographic and health-related characteristics among the individuals in the intervention group and the usual care group using Student *t* test, Wilcoxon signed rank test, chi-square test, and Fisher exact test, as appropriate. We calculated the change in outcomes over time as the difference between baseline and 3 months and baseline and 9 months for diabetes self-efficacy scores, depression severity (PHQ-9), stress score, and PROMIS measures. We used Student *t* test to compare the change in outcome between the usual care and intervention groups (significance level: *P*≤.05). We conducted statistical analysis using Stata, version 15.0, statistical software (StataCorp, Texas, US).

In the primary analysis, we estimated the difference over time in the effects of the intervention versus usual care in the study participants, controlling for potentially relevant variables such as demographic characteristics, readiness to change, self-reported health, and comorbid disease. This was an intention-to-treat analysis with the assumption that any dropouts were missing at random. In our evaluation, we did not find any significant difference between the participants who dropped out and the participants who continued in the study. We adopted a mixed effects maximum likelihood model that accounts for the missing data from the participants who dropped out from the study. Finally, our sensitivity analysis found no difference with regard to the significance of our findings when we excluded these participants from the analysis. We included all participants, regardless of intervention completion, in the intention-to-treat analysis. We used multivariate regression modeling for all hypotheses testing to estimate within-group and across-group effects of the intervention on the outcomes (significance level: *P*≤.05). The mixed effects models evaluated the impact of the intervention over time on the primary outcome, diabetes self-efficacy. We included a binary indicator for intervention group assignment and a group-by-time interaction term in the models to compare improvement over time between the intervention group and usual care group. We evaluated model fit using deviance tests for nested models, and the Akaike information criterion and the Bayesian information criterion for non-nested models. We assessed the estimates for the fixed effects using a predetermined significance level (*P*<.05) on 2-sided tests and 95% CIs. We used the same approach for analyzing the primary and secondary outcomes, analyzing the effect of the intervention at baseline, 3 months, and 9 months.

## Results

### Overview

Multimedia Appendix 2 provides a Consolidated Standards of Reporting Trials (CONSORT) flow diagram. The diabetes registry query identified 2242 potential participants. Of these, 1938 were eligible for phone recruitment. We obtained verbal consent from 392 participants by phone before randomization. A total of 319 participants attended the orientation session, completed the written consent, and were included in the analysis. Furthermore, 32 out of 319 (10.0%) participants, 6 out of 161 (3.7%) participants from the usual care group and 26 out of 158 (16.5%) participants from the intervention group, either dropped out or were lost to follow-up over the course of the study. Of the 287 participants who completed the 9-month follow-up surveys, 155 were in the usual care group and 132 were in the intervention group. The recruitment commenced in February 2016 and the study was completed by December 2017.

### Sample Characteristics

There were no significant differences in the demographics between the usual care and intervention groups (Table 1), with an almost equal distribution in gender and a mean age of 59.1 (SD 11.4) years. Most participants identified themselves as white, followed by African American, Asian, other, or more than 1 race. Furthermore, 42 out of 277 (15.2%) participants identified themselves as Hispanic and Latino. The majority of participants had attained at least some college education or completed college degrees.

Most participants were managing multiple chronic illnesses. The participants tracked metrics related to their health at varying rates as follows: blood glucose (127/319, 39.8%), laboratory results (125/319, 39.2%), physical activity (82/319, 25.7%), nutrition (65/319, 20.4%), and sleep (54/319, 16.9%). A large percentage of participants, 256 out of 319 (80.3%), had no prior experience using mobile apps or sensors. The usual care and intervention groups were similar with regard to their baseline health characteristics and experience with technology.

**Table 1:** Characteristics of participants in the study.

Characteristics		Total (N=319)	Control (n=161)	Intervention (n=158)	*P* value
**Gender, n (%) (N=313)**					.97
	Female	148 (47.3)	75 (47.2)	73 (47.4)	
	Male	165 (52.7)	84 (52.8)	81 (52.6)	
Age (years), mean (SD)		59.07 (11.4)	59.18 (11.5)	58.96 (11.3)	.87
**Education, n (%) (N=315)**					.89
	High school or less	36 (11.4)	20 (12.6)	16 (10.3)	
	Some college	109 (34.6)	56 (35.2)	53 (34.0)	
	Associate’s degree	40 (12.7)	19 (12.0)	21 (13.5)	
	Bachelor’s degree	63 (20.0)	29 (18.2)	34 (21.8)	
	Graduate and professional degree	67 (21.3)	35 (22.0)	32 (20.5)	
**Annual income, n (%) (N=276)**					.06
	<US $25,000	44 (15.9)	29 (21.3)	15 (10.7)	
	US $25,000-US $50,000	66 (23.9)	29 (21.3)	37 (26.4)	
	US $50,001-US $75,000	56 (20.3)	29 (21.3)	27 (19.3)	
	US $75,001-US $100,000	47 (17.0)	17 (12.5)	30 (21.4)	
	>US $100,000	63 (22.8)	32 (23.5)	31 (22.1)	
**Race, n (%) (N=311)**					.79
	Caucasian	196 (63.0)	100 (62.9)	96 (63.2)	
	African American	39 (12.5)	18 (11.3)	21 (13.8)	
	Asian	27 (8.7)	16 (10.1)	11 (7.2)	
	Other	30 (9.7)	14 (8.8)	16 (10.5)	
	More than 1 race	19 (6.1)	11 (6.9)	8 (5.3)	
**Ethnicity, n (%) (N=277)**					.28
	Hispanic or Latino	42 (15.2)	18 (12.9)	24 (17.5)	
	Not Hispanic or Latino	235 (84.8)	122 (87.1)	113 (82.5)	
**Chronic comorbidities, n (%) (N=307)**					.88
	No other comorbidities	115 (37.5)	55 (34.2)	60 (38.0)	
	1 comorbidity	95 (30.9)	47 (29.2)	48 (30.4)	
	2 comorbidities	52 (16.9)	27 (16.8)	25 (15.8)	
	3 or more comorbidities	45 (14.7)	25 (15.5)	20 (12.7)	
**Experience with health apps or sensors, n (%) (N=317)**					.74
	Previously used apps or sensors	63 (19.8)	33 (20.5)	30 (19.0)	
	Never used apps or sensors	256 (80.3)	128 (79.5)	128 (81.0)	
**Current health tracking, n (%)**					
	Blood glucose	127 (39.8)	64 (39.8)	63 (39.9)	.31
	Physical activity	82 (25.7)	35 (21.7)	47 (29.8)	.25
	Nutrition	65 (20.4)	33 (20.5)	32 (20.3)	.46
	Sleep	54 (16.9)	28 (17.4)	26 (16.5)	.42

### Intervention Engagement

The most common smart goals selected by the intervention group participants were physical activity (103/147, or 70.7%) and nutrition (37 out of 147 or 25.2%), with 7 out of 147 (4.8%) participants selecting other goals such as stress reduction, alcohol cessation, and improving sleep. Across all coaching sessions, participants averaged 172 min of nurse coaching.

### Study Outcomes

Multimedia Appendices 3 and 4 summarize the descriptive results for the study outcomes. At baseline, the mean diabetes self-efficacy score was 3.66 (SD 0.89) in the usual care group and 3.67 (SD 0.83) in the intervention group. This score increased in both groups at 3 months, 3.71 (SD 0.86) in the usual care group and 4.05 (SD 0.69) in the intervention group. For the depression severity measure, PHQ-9, the usual care group experienced slightly greater depressive symptoms over time, whereas the intervention group’s PHQ-9 score decreased at 3 months. There were no changes in the perceived stress or the PROMIS measures. For the intervention group at baseline, the average number of steps per week was 23,770 (SD 18,470), which increased at 3 months to 39,167 (SD 22,513) and declined at 9 months to 32,601 (SD 19,851). Furthermore, 82 of the 132 (62.1%) participants who completed the 9-month survey continued to use the device until the end of the study.

Multimedia Appendices 5 and 6 show the comparison of changes in outcomes (difference in differences) between the participants in the intervention group and the participants in the usual care group. The analysis of outcome measures at baseline and 3 months demonstrated a significant improvement in diabetes self-efficacy (DES), 0.34 (95% CI –0.15,0.53), and depressive symptoms (PHQ-9), 0.89 (95% CI 0.01-1.77), and a trend toward decreased perceived stress (0.59, 95% CI 0.03-1.16) in the intervention group compared with the usual care group. There were no significant differences in emotional distress, anxiety, or physical functioning (PROMIS) at different time intervals. There were no significant differences in the outcome measures at baseline and 9 months (end of study) between participants in the intervention and usual care groups.

Finally, our mixed effect regression models evaluated the effect of the intervention on the primary outcome, diabetes empowerment (DES), over time (3 months and 9 months). We found significant improvement in the DES scores at 3 months (0.50, 95% CI 0.07-1.1; *P*<.05) in the intervention group, but this was not sustained at 9 months after adjusting for readiness to change, self-reported health, gender, education, race, and comorbid disease in the final model. We did not find significant changes in the PHQ-9 and PSS scores after adjusting for other factors in the regression models.

## Discussion

### Principal Findings

This study built upon the recognition that chronic disease management is fundamentally a partnership between health care providers and individuals, requiring goal setting, bilateral communication, and motivation. We sought to change the conversation through mHealth technology and nurse coaches’ support that could standardize goal setting and generate relevant patient-generated data for discussion and action. We engaged the stakeholders representing persons with diabetes, clinicians, and health information technology experts to design the intervention and the integration of the technology and coaching into primary care.

This study demonstrated the short-term effectiveness of an innovative diabetes intervention using nurse health coaching and mHealth technology on diabetes self-efficacy and increased physical activity, supporting our hypothesis. However, by 6 months post intervention, although physical activity remained above baseline, the differences in self-efficacy were not sustained.

The change in diabetes self-efficacy score represented a meaningful improvement in the confidence for engaging in self-management behavior in 3 to 4 out of 8 potential areas. The average PHQ-9 score was below the cutoff indicating substantial depressive symptoms, and the score decreased over the course of the intervention. Improved self-efficacy and management of depression may be the most important drivers for positive health behavior change. This finding also suggests a potential for PGHD derived from wearable sensors to play a role in improving self-efficacy. By using tracking devices, the participants became aware of their physical activity and behavior patterns and could visualize and measure their accomplishments, increasing motivation to continue changes to attain their goals. The nurse coaches explaining the data from sensors and supporting the participants as personal difficulties arose were critical in the positive outcomes as the coaching interactions also created accountability, focus, and awareness of how behavior impacted the participants’ health.

The decrease in depression severity scores among the intervention group participants further endorse the importance of personalized support, connection to health care resources to cope with and treat depression, and continuous communication provided during the coaching sessions. Significant differences in these areas did not persist at 9 months. As lifestyle changes take time and require reinforcement, it is possible that a longer intervention period with continuing support (ie, health coaching) could be needed for sustained behavior changes leading to better diabetes management.

Participants in the intervention demonstrated a significant increase in physical activity, as measured by increased steps per week, from 23,700 to 39,167 at 3 months and down to 32,601 at 9 months. Using average stride length, this is an increase from about 11.9 miles to 19.6 miles walked per week. At 9 months, participants were still walking 16 miles. This accomplishment is consistent with the Centers for Disease Control and Prevention’s (CDC’s) recommended guidelines for type 2 diabetes management to get at least 150 min per week of moderate-intensity physical activity [36] and indicates an improvement in lifestyle choices. According to the CDC, incremental and sustained improvements in physical activity over time will contribute to better management of type 2 diabetes.

The lack of long-term sustainability of this intervention echoes other studies [37-39]. In a hospital-based telephone coaching intervention for patients with type 2 diabetes, Varney et al [37] reported that the intervention was only effective during the study period but not sustainable past the completion of the study. Mohr et al [39] and Yardley et al [38] discussed the importance of human support in motivation, effective engagement, and adherence of participants to electronic health interventions.

The high retention rate (89.9% completed the 9-month study), coupled with the qualitative feedback from participants, suggests that the intervention was acceptable and useful despite the fact that over 80% had no previous experience with technology. Both groups engaged with the technology; the usual care group demonstrated interest and use of online diabetes resources, and the intervention group used their wearable activity trackers and online resources. Although the digital divide exists, this study demonstrates that mHealth solutions are viable for novices to technology and individuals with low socioeconomic status. Fu et al [40] identified specific elements that could enhance the utilization of diabetes apps, including assisting participants to recognize patterns, customizing targets, reviewing data, and planning lifestyle adjustments.

### Strengths and Limitations

The passive tracking devices provided data for discussion with the nurse coaches who assisted in making sense of the patterns, generating insights into health habits that can affect outcomes that matter to the patient. These insights form the basis for long-term behavior change necessary for optimal health in diabetes. In a parallel analysis of the qualitative data from this study, participants described new insights about living with type 2 diabetes and defined success in their own terms as changes in awareness, mindset, engagement with health resources, and self-perceptions of emotional or physical health [41]. Participants in our trial substantiated the importance of context, meaning, and health care partnerships in engaging and sustaining engagement with the use of mHealth technology [19].

This study also demonstrated the feasibility of integrating such PGHD into the EHR for visualization by the patient and the health care team. Although the use of mHealth to improve diabetes self-management is well illustrated in the research literature [42-44], the data gathered in those studies were only used for research, without integration into the patient’s EHR for optimal utilization by clinicians managing the disease. The EHR typically stores data generated by providers and the health system, with no contribution of data either generated by the patient or deemed important by patients in creating a complete picture of their health. To our knowledge, this is the first study reporting successful comprehensive integration of PGHD not only into the EHR at an academic medical center but also its meaningful integration into the provider’s workflow.

This study had several limitations. First, the sample might have been biased toward those ready to change and those ready to use technology, limiting generalizability. Second, this study has limited generalizability to other settings because of required investments in technology, technology training, and support throughout the intervention. However, we estimated the total cost of the intervention, including staff time and technology, to be less than US $500 per participant. The total cost is a small investment relative to the costs of an office or emergency room visit. Finally, this intervention requires commitment to a model of care to include nurse coaches in a team-based approach.

Although this study demonstrated the short-term effects on self-efficacy, it did not demonstrate sustainability of the effects. Since this study commenced, 2 major trends have accelerated—the use of mHealth technology and the movement toward value-based payment for chronic disease management. These facilitating forces will likely result in approaches akin to those examined in this study. This study answered several logistical questions for scalability and generalizability, demonstrated the feasibility of integrating PGHD into the clinical record, and engaged and supported patients with little previous exposure to technology. As mobile technology is gaining adoption across all age demographics [45], scalability becomes more reasonable with more tech-savvy patients who already own mobile phones. EHR vendors are now working on HIPPA-compliant platforms that incorporate PGHD. With this forward movement in technology, the intervention becomes even more feasible.

Although traditional fee-for-service reimbursement models would not cover such interventions, managed care plans and value-based purchasing will move toward reimbursing interventions that yield quality outcomes, further enhancing the potential for sustaining interventions of this kind. Finally, as PGHD are not highly sensitive, this intervention does not have to be clinic-based. It has the potential for delivery in a variety of settings such as fitness centers, workplaces, and community centers, increasing the potential to scale.

### Conclusions

Coupling patient data with other indicators of health provides a more complete picture that could be helpful in a variety of chronic conditions, such as congestive heart failure and chronic obstructive pulmonary disease, which require a partnership between clinicians and patients for effective management. This study combined nurse coaching with mHealth and compared this intervention with usual care; future studies could include more study arms that break out the components to allow greater comparison. Future research could examine this approach in additional conditions and other strategies, such as automated feedback in the form of SMS messaging and online peer support that could enhance the effectiveness of the intervention. Longer study periods with intermittent coach contact could potentially demonstrate how to sustain the effects of the intervention over time. Finally, studies of implementation and dissemination across a variety of settings would improve the translation of research such as this into practice.

## Appendix

Multimedia Appendix 1 Intervention figure.

Multimedia Appendix 2 CONSORT flow diagram.

Multimedia Appendix 3 Outcome measures at baseline, 3 months, and 9 months for control group table.

Multimedia Appendix 4 Outcome measures at baseline, 3 months, and 9 months for intervention group table.

Multimedia Appendix 5 Change in outcomes comparing baseline and 3 months (Difference in Difference).

Multimedia Appendix 6 Change in outcomes comparing baseline and 9 months (Difference in Difference) table.

Multimedia Appendix 7 CONSORT-EHEALTH checklist (V 1.6.1).

## Abbreviations

CDC: Centers for Disease Control and Prevention
DES: Diabetes Empowerment Scale
EHR: electronic health record
mHealth: mobile health
MI: motivational interviewing
MITI: Motivational Interviewing Treatment Integrity
P^2^E^2^T^2^: Patient and Provider Engagement and Empowerment Through Technology
PGHD: patient-generated health data
PHQ-9: Patient Health Questionnaire-9
PROMIS: Patient-Reported Outcomes Measurement Information System
PSS: Perceived Stress Scale
RHC: Registered Health Coach
RN: registered nurse
