# Supplementary material for: Nurse Coaching and Mobile Health Compared With Usual Care to Improve Diabetes Self-Efficacy for Persons With Type 2 Diabetes: Randomized Controlled Trial
Source: JMIR Mhealth Uhealth. 2020 Mar 2;8(3):e16665. doi: 10.2196/16665 (PMC7076411; doi:10.2196/16665)
Supplement: Multimedia Appendix 1 [file mhealth_v8i3e16665_app1.docx]

Multimedia Appendix 1:

Intervention Figure

Orient participant to study and set up participant with technology [for usual care: orientation to existing on-line resources and for intervention arm: tracking device, iPod if necessary, applications (MyChart; MFP; Basis/Garmin)]

Initial coaching telephone call with participant and nurse coach –set initial goals , troubleshoot technology, review of initial data integrated into PGHD module (all data elements)

Coaching telephone calls #2-5 with participant & nurse coach occurring every 2 weeks with bi-weekly review of shared health goals & data element streams integrated into PGHD module to measure goal success (data elements chosen by participant), and technology troubleshooting. Through discussion, using motivational interviewing techniques, participants were encouraged to identify facilitators for success and barriers that prevented them from achieving their goals. Nurse coaches encouraged exploration through open-ended questions and offering gentle prompts and encouragement.

Final coaching telephone call with participant and nurse coach - reviewed overall goals and goal success as determined by participant and review of PGHD elements in EHR

Final summary report by nurse coach sent to participant’s primary care provider with health goals and overall goal success
