# Supplementary figures and images for: Nurse Coaching and Mobile Health Compared With Usual Care to Improve Diabetes Self-Efficacy for Persons With Type 2 Diabetes: Randomized Controlled Trial
Source: JMIR Mhealth Uhealth. 2020 Mar 2;8(3):e16665. doi: 10.2196/16665 (PMC7076411; doi:10.2196/16665)

Multimedia Appendix 2:

CONSORT Flow Diagram


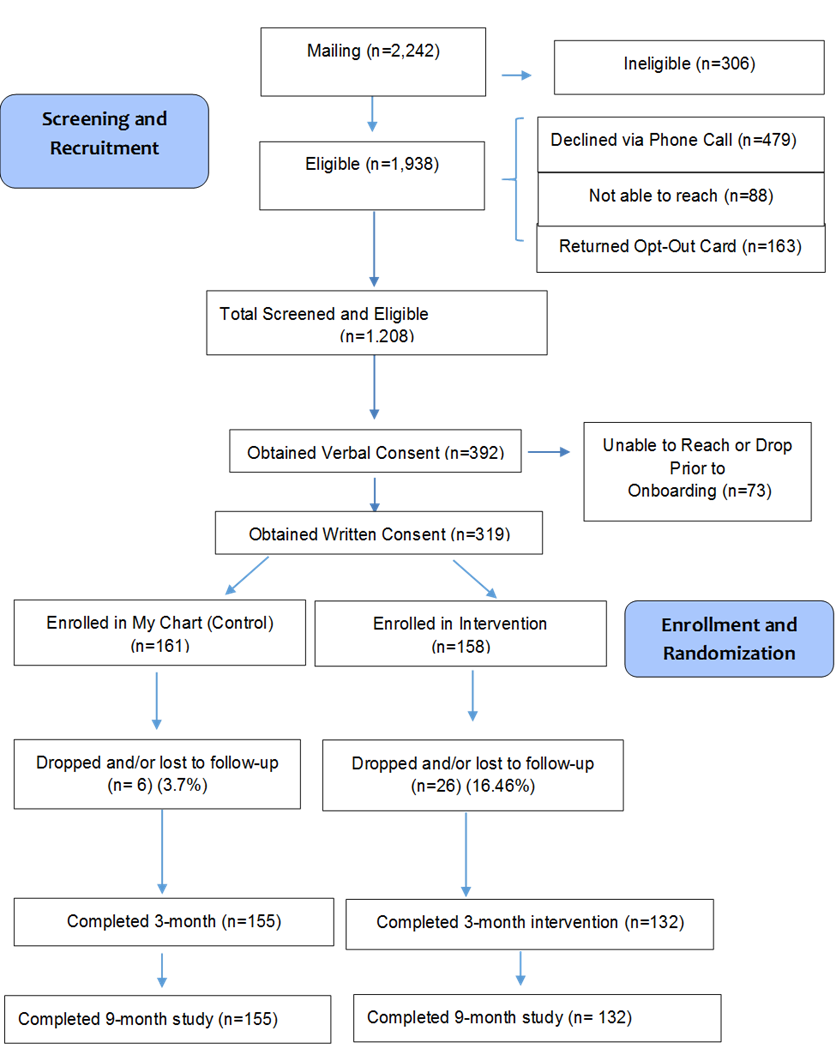

Supplement: Multimedia Appendix 2 [file mhealth_v8i3e16665_app2.docx]
