# Supplementary material for: Nurse Coaching and Mobile Health Compared With Usual Care to Improve Diabetes Self-Efficacy for Persons With Type 2 Diabetes: Randomized Controlled Trial
Source: JMIR Mhealth Uhealth. 2020 Mar 2;8(3):e16665. doi: 10.2196/16665 (PMC7076411; doi:10.2196/16665)
Supplement: Multimedia Appendix 3 [file mhealth_v8i3e16665_app3.docx]

Multimedia Appendix 3:

Outcomes measures at baseline, 3 months, and 9 months for control group table

| *Outcomes, mean (SD)* | *Control group* | | |
| --- | --- | --- | --- |
|  | Baseline | 3 months | 9 months |
| *Primary Outcome* | | | |
| *Diabetes self-efficacy* | 3.66 (.89) | 3.71 (.86) | 3.95 (.97) |
| *Secondary Outcomes* | | | |
| *Depression severity (PHQ9)* | 5.36 (4.64) | 5.44 (5.24) | 5.69 (6.58) |
| *Perceived stress (PSS)* | 4.98 (3.08) | 4.98 (2.99) | 5.37 (3.48) |
| *Other Outcomes* | | | |
| *PROMIS Emotional distress anxiety* | 51.96 (9.98) | 49.10 (9.58) | 51.46 (9.00) |
| *PROMIS Physical functioning* | 30.09 (8.04) | 29.79 (7.51) | 29.34 (8.27) |
| *Number steps/week* | - | - | - |
