# Supplementary material for: Nurse Coaching and Mobile Health Compared With Usual Care to Improve Diabetes Self-Efficacy for Persons With Type 2 Diabetes: Randomized Controlled Trial
Source: JMIR Mhealth Uhealth. 2020 Mar 2;8(3):e16665. doi: 10.2196/16665 (PMC7076411; doi:10.2196/16665)
Supplement: Multimedia Appendix 4 [file mhealth_v8i3e16665_app4.docx]

Multimedia Appendix 4:

Outcomes measures at baseline, 3 months, and 9 months for intervention group tables

| *Outcomes, mean (SD)* | *Intervention group* | | |
| --- | --- | --- | --- |
|  | Baseline | 3 months | 9 months |
| *Primary Outcome* | | | |
| *Diabetes self-efficacy* | 3.67 (.83) | 4.05 (.69) | 3.97 (.91) |
| *Secondary Outcomes* | | | |
| *Depression severity (PHQ9)* | 5.00 (4.99) | 4.19 (4.51) | 4.81 (5.26) |
| *Perceived stress (PSS)* | 5.24 (2.99) | 4.64 (3.05) | 5.22 (3.05) |
| *Other Outcomes* | | | |
| *PROMIS Emotional distress anxiety* | 53.05 (10.10) | 49.39 (8.88) | 52.89 (10.64) |
| *PROMIS Physical functioning* | 29.23 (6.01) | 29.26 (6.56) | 29.77 (7.28) |
| *Number steps/week* | 23,770 (18,470) | 39,167 (22,513) | 32,601 (19,851) |
