# Supplementary material for: Nurse Coaching and Mobile Health Compared With Usual Care to Improve Diabetes Self-Efficacy for Persons With Type 2 Diabetes: Randomized Controlled Trial
Source: JMIR Mhealth Uhealth. 2020 Mar 2;8(3):e16665. doi: 10.2196/16665 (PMC7076411; doi:10.2196/16665)
Supplement: Multimedia Appendix 5 [file mhealth_v8i3e16665_app5.docx]

Multimedia Appendix 5:

Change in outcomes comparing baseline and 3 months (Difference in Difference) table

|  | *Baseline and 3 months* | | | |
| --- | --- | --- | --- | --- |
|  | Control | Intervention | Difference (95% CI) | *P*-value |
| *Primary Outcome* | | | | |
| Diabetes self-efficacy^a^ | .05  (-.09,.19) | .39  (.26, .51) | .34  (-.15, .53) | .00 |
| *Secondary Outcomes* | | | | |
| Depression severity ^b^ | .09  (-.58,.75) | -.81  (-1.38, .23) | .89  (.01, 1.77) | .05 |
| Perceived stress scale^b^ | 0  (-.42,.40) | -.60  (-.99, -.21) | .59  (.03,1.16) | .04 |
| *Other Outcomes* | | | | |
| Emotional distress anxiety^b^ | -2.87  (-4.83,.90) | -3.66  (-5.74, 1.57) | .79  (-2.10,3.65) | .6 |
| Physical functioning^a^ | -.29  (-1.77,1.18) | .03  (-1.41, 1.47) | -.33  (-2.40,1.75) | .8 |

^a^ higher score is better ^b^ lower score is better
