# Supplementary material for: Nurse Coaching and Mobile Health Compared With Usual Care to Improve Diabetes Self-Efficacy for Persons With Type 2 Diabetes: Randomized Controlled Trial
Source: JMIR Mhealth Uhealth. 2020 Mar 2;8(3):e16665. doi: 10.2196/16665 (PMC7076411; doi:10.2196/16665)
Supplement: Multimedia Appendix 6 [file mhealth_v8i3e16665_app6.docx]

Multimedia Appendix 6:

Change in outcomes comparing baseline and 9 months (Difference in Difference) table

|  | *Baseline and 9 months* | | | |
| --- | --- | --- | --- | --- |
|  | Control | Intervention | Difference (95% CI) | *P*-value |
| *Primary Outcome* | | | | |
| Diabetes self-efficacy^a^ | .29  (.11, .46) | .31  (.15, .46) | .02  (.28,.24) | .9 |
| *Secondary Outcomes* | | | | |
| Depression severity ^b^ | .33  (-.50, 1.16) | -.34  (-1.13, .44) | .67  (-.47,1.82) | .2 |
| Perceived stress scale^b^ | .39  (-.11, 0.88) | -.02  (-.45, .42) | .41  (-.14,1.06) | .2 |
| *Other Outcomes* | | | | |
| Emotional distress anxiety^b^ | -.51  (-2.55, 1.54) | -.16  (-2.77, 2.45) | -.35  (-3.60,2.91) | .8 |
| Physical functioning^a^ | -.75  (-2.36, .86) | .54  (-1.02, 2.09) | -1.29  (-3.54,.97) | .3 |

^a^ higher score is better ^b^ lower score is better
